# Nickel ferrite decorated noble metal containing nitrogen-doped carbon nanotubes as potential magnetic separable catalyst for dinitrotoluene hydrogenation

**DOI:** 10.1038/s41598-024-66066-1

**Published:** 2024-07-02

**Authors:** Viktória Hajdu, Emőke Sikora, Gábor Muránszky, Ferenc Kristály, Zoltán Kaleta, Miklós Nagy, Béla Viskolcz, Béla Fiser, László Vanyorek

**Affiliations:** 1 Institute of Chemistry, University of Miskolc, Miskolc-Egyetemváros, 3515 Hungary; 2 Higher Education and Industrial Cooperation Centre, University of Miskolc, Miskolc-Egyetemváros, 3515 Hungary; 3 Institute of Mineralogy and Geology, University of Miskolc, Miskolc-Egyetemváros, 3515 Hungary; 4 Department of Organic Chemistry, Semmelweis University, Budapest, 1092 Hungary; 5 Ferenc Rakoczi II Transcarpathian Hungarian College of Higher Education, Beregszász, 90200 Ukraine; 6 Department of Physical Chemistry, Faculty of Chemistry, University of Lodz, 90-236 Lodz, Poland

**Keywords:** Catalytic reactions, Magnetic catalyst, Polyurethane precursor, Polymer industry, Toluenediamine, Chemistry, Catalysis, Chemical synthesis

## Abstract

The 2,4-toluenediamine (TDA) is one of the most important chemicals in the polyurethane industry, produced by the catalytic hydrogenation of 2,4-dinitrotoluene (DNT). The development of novel catalysts that can be easily recovered from the reaction mixture is of paramount importance. In our work, a NiFe_2_O_4_/N-BCNT supported magnetic catalyst was prepared by a modified coprecipitation method. The catalyst support alone also showed activity in the synthesis of TDA. Platinum nanoparticles were deposited on the catalyst support surface by a fast, relatively simple, and efficient sonochemical method, resulting in a readily applicable catalytically active system. The prepared catalyst exhibited high activity in hydrogenation tests, which was proved by the exceptionally high DNT conversion (100% for 120 min at 333 K) and TDA yield (99%). Furthermore, the magnetic catalyst can be easily recovered from the reaction medium by the action of an external magnetic field, which can greatly reduce catalyst loss during separation.

## Introduction

Toluene diamine (TDA) is one of the most important raw materials in polyurethane (PU) production, with the global TDA market size was $1.89 billion in 2023. TDA is produced by the catalytic hydrogenation of dinitrotoluene (DNT) in the presence of a catalyst in the liquid phase. Several different catalysts have been successfully used for dinitrotoluene hydrogenation, such as Ni, Fe, Pd, Pt, Ir or bimetallic systems supported on carbon (C), alumina (Al_2_O_3_) or silica (SiO_2_)^1–6^. During the process, several intermediate reactions occur. As intermediate products, 2-(hydroxyamino)-4-nitrotoluene (2HA4NT), 4-(hydroxyamino)-2-nitrotoluene (4HA2NT), 2-amino-4-nitrotoluene (2A4NT), and 4-amino-2-nitrotoluene (4A2NT) can be formed.

Among carbon modifications, activated carbon is the most widespread, but the application of nanostructured carbon forms, primarily carbon nanotubes, is becoming increasingly characteristic. The reason for their popularity is that they possess impressive properties, including exceptional mechanical strength, good chemical stability, and high specific surface area, making them ideal catalyst supports^7^. The characteristics of carbon nanotubes (CNTs) can be further modified by incorporating heteroatoms into their structure^8^. The synthesis of CNTs using nitrogen-containing carbon compounds as starting materials leads to the formation of N-doped carbon nanotubes (which is also called nitrogen-doped bamboo-shaped carbon nanotubes, N-BCNTs)^9^. Due to the additional binding sites a higher amount of catalytically active metal particles can be bound to their surface, which in turn leads to a higher catalytic activity in a reaction^10,11^. Carbon nanotube-supported catalysts are characterized by their excellent dispersibility in liquid reaction media, which is advantageous in the catalytic process, as it allows for a larger number of catalytic noble metal nanoparticles to be reached on the surface of the carbon nanotubes, where the hydrogenation reaction takes place. However, at the end of the reaction, the catalyst needs to be recovered from the reaction medium, which is not easy for a stable dispersion. In the case of nanostructured catalysts, this requires some separation step (filtration, centrifugation), which is not efficient due to the small particle size^12^. Nanoparticles with magnetic properties (iron oxides, ferrites) have the particle size and surface polarity to ensure adequate dispersion stability during the hydrogenation process^13,14^. Another major advantage is that they can be separated from the liquid phase by a magnetic field, allowing the catalyst to be easily recovered after the synthesis^15,16^. A similar magnetically recoverable system has been prepared by Ma and co-workers, the Co@C/CNTs-10-700 catalyst showed excellent conversion and selectivity for transfer hydrogenation of nitroarenes with different hydrogen donors. Moreover, the magnetic property of the catalyst allowed it to be reused in up to ten consecutive cycles without significant loss of activity^17^. Shokouhimehr and co-workers synthesized nanocomposite catalysts based on carbon and polypyrrole, respectively, which resulted in enhanced catalytic activity in the selective reduction of nitroarenes and Suzuki cross-coupling reactions. The synthesized nanocomposite catalysts can be easily separated from the reaction mixture by means of a magnet and can be reused repeatedly in nitrobenzene reduction and Suzuki cross-coupling without significant loss of activity^18,19^. Several studies have been carried out to understand the nature of the synergistic effects between NiFe_2_O_4_ magnetic nanoparticles and carbon nanotubes^20,21^. These combined magnetic systems which include carbon nanotubes have been widely used in catalysis^22^, electronic devices^23^, biosensors, and for antitumor and anticancer activities^24^.

In this research, a Pt/NiFe_2_O_4_/N-BCNT magnetic hydrogenation catalyst was prepared by a modified coprecipitation method and tested its applicability in the hydrogenation of 2,4-dinitrotoluene. The nitrogen-doped nanostructured carbon forms which were employed are remarkable catalyst supports, as the incorporated nitrogen atoms modify the electron distribution of the carbon structure, and this contributes to a stronger interaction between the catalytically active metal and the support. They also contribute to improve the catalytic activity of the catalyst through electron transfer between the metal and the electronegative atoms of the support (e.g. C, N, O). These positive features can be further enriched by the introduction of magnetic properties. The developed nitrogen doped carbon supported platinum catalysts are decorated with nickel-ferrite nanospheres, allowing them to be easily separated from the liquid phase after the reaction by means of a magnet. Thus, due to the magnetic behaviour, the conventional time-consuming separation steps (filtration, centrifugation) can be avoided. The performance of the catalyst was further compared to other catalytic systems in dinitrotoluene hydrogenation by using the MIRA21 database^25^.

## Materials and methods

### Materials

The N-BCNT was produced by catalytic chemical vapor deposition (CCVD) (https://www.sciencedirect.com/topics/materials-science/chemical-vapor-deposition) method from nbutylamine (C_4_H_11_N, Merck KGaA, D-64271 Darmstadt, Germany) as carbon source. As catalyst of CCVD synthesis of the carbon nanotubes was used nickel(II) nitrate hexahydrate (Ni(NO_3_)_2_ ∙6 H_2_O, MW:290.79 g/mol, Thermo Fisher GmbH, D-76870 Kandel, Germany) was employed as catalyst during the CCVD synthesis, impregnated magnesium oxid (MgO, Merck KGaA, D-64271 Darmstadt, Germany). To prepare the nickel ferrite particles iron(III) nitrate nonahydrate (Fe(NO_3_)_3_∙9H_2_O, MW:404.00 g/mol VWR Int. LtD., B-3001 Leuven, Belgium), ethylene glycol, (HOCH_2_CH_2_OH, VWR Int. Ltd., F-94126 Fontenay-sous-Bois, France), ethanolamine (NH_2_CH_2_OH, Merck KGaA, D-64271 Darmstadt, Germany) and sodium acetate (CH_3_COONa, ThermoFisher GmbH, D-76870 Kandel, Germany) were used. Dihydrogen hexachloroplatinate(IV) hydrate (H_2_PtCl_6_∙H_2_O, Alfa Aesar Ltd., Ward Hill, MA, USA), hydrazine hydrate (N_2_H_4_ x H_2_O, Sigma Aldrich Chemie Gmbh, D-89555 Steinheim, Germany) and patosolv (a mixture of 90 vol% ethanol and 10 vol% isopropanol, Molar Chem. Ltd., H-2314 Halásztelek, Hungary) were used to prepare platinum nanoparticles. Catalytic tests were carried out using 2,4-dinitrotoluene (C_7_H_6_N_2_O_4_, Sigma Aldrich Chemie Gmbh, D-89555 Steinheim, Germany), nitrobenzene (C_6_H_5_NO_2_, Merck KGaA, D-64293 Darmstadt, Germany), methanol (Molar Chem. Ltd., H-2314 Halásztelek, Hungary) and hydrogen gas (5.0 purity, Messer Ltd.).

### Synthesis of the NBCNTs

N-BCNT synthesis was carried out by using CCVD method. During the CCVD synthesis 5 wt% nickel containing magnesium oxide (as catalyst) was placed into the quartz reactor in tube furnace, which was heated at 1023 K temperature. The carbon source (butylamine) was injected by a syringe pump (16 ml h^−1^) into the reactor, nitrogen (50 ml min^−1^) flow carried the vapor into the catalyst bed. The nickel containing catalyst was removed by hydrochloride acid from the nanotubes. The purity of N-BCNTs was measured by thermogravimetric analysis, and it was 98.5 wt%.

### Deposition of the nickel ferrite nanoparticles on the surface of the N-BCNTs

The magnetic separable nickel ferrite loaded N-BCNTs (as catalyst support) were functionalized by amine groups and applied in a modified coprecipitation method. In the first step, 32.32 g Fe(NO_3_)_3_∙9 H_2_O and 11.63 g Ni(NO_3_)_2_∙6 H_2_O precursors were dissolved (reactant I.) in 400 ml ethylene glycol.

Dispersion of N-BCNT (reactant II) was also prepared, by using 2.00 L ethylene glycol within which 49.22 g sodium acetate was dissolved and thereafter, in this solution 10.00 g nanotubes was dispersed by ultrasound agitation (by Hielscher UIP 1000 hDT ultrahigh efficient homogenizer). The dispersion of nanotubes (reactant II) was heated to 100 °C in round bottom flask under reflux with continuous stirring. The solution of the iron(III)- and nickel(II)-precursors (reactant I) was added into the dispersion, and after that, 140 ml ethanol amine was also dosed. After 12 h of continuous agitation and reflux, the cooled solution was separated by centrifugation (4200 rpm, at 10 min). The ethylene glycol and the remaining reactants were eliminated by washing with water from the nanocomposite. Finally, the ferrite decorated carbon samples were rinsed by ethanol, and were dried at 80 °C, overnight. This ferrite containing samples were used as magnetic catalyst support for the preparation of platinum decorated spinel catalysts.

### Deposition of the platinum nanoparticles on the surface of the ferrite decorated N-BCNTs

The magnetizable ferrit containing N-BCNT (2.00 g) was dispersed in 200 mL ethanol by ultrasonication with Hielscher UIP1000 hDT ultrasound system. Dihydrogen hexachloroplatinate(IV) was dissolved in 50 ml ethanol and 1 mL hydrazine hydrate was also used, and it was added to the alcoholic dispersion of NiFe_2_O_4_/N-BCNT and treated by ultrasonic agitation for 10 min. The final Pt/ NiFe_2_O_4_/N-BCNT catalysts were separated by magnetic field from the ethanolic phase, and the catalyst was dried at 378 K overnight.

### Characterization technics

The catalysts were examined by high-resolution transmission electron microscopy (HRTEM, FEI Technai G2 electron microscope, 200 kV) for characterization of morphology, particle size and structure of the nickel-ferrite and platinum nanoparticles. For the electron diffraction a SmartCam digital search camera (Ceta 16 Mpixel, 4 k × 4 k CMOS camera) and a high-angle annular dark-field (HAADF) detector were used. The specimens were prepared by dropping aqueous suspension of the catalysts on 300 mesh copper grids (Ted Pella Inc.). The identification of the crystalline phases was carried by X-ray diffraction (XRD) measurements by using a Bruker D8 Advance diffractometer (Cu-Kα source, 40 kV and 40 mA) in parallel beam geometry (Göbel mirror) with Vantec1 detector. Functional groups on the surface of the nanotube support were studied by using Fourier-transform infrared spectroscopy (FTIR, Bruker Vertex 70 spectrometer). The sample (10 mg) was pelletized with potassium bromide (250 mg) and the IR spectrum was made in transmission mode. The X-ray photoelectron spectroscopy (XPS) measurements were performed by a SPECS instrument equipped with a PHOIBOS 150 MCD nine analyzer. The Al Kα x-ray source was operated at 14 kV and 10.8 mA (150W). The analyzer was operated in FAT mode with a pass energy of 20 eV. High resolution spectra were acquired by averaging 15 spectra of each region. The CasaXPS software was used for data evaluation.

The specific surface area (SSA) of the catalyst was measured by CO_2_ adsorption–desorption experiments at 273 K by using a Micromeritics ASAP 2020 sorptometer based on the Dubinin–Astakhov (DA) method. The metal content of the magnetic catalyst was analysed by a Varian 720 ES inductively coupled optical emission spectrometer (ICP-OES) using Merck Certipur ICP multi-element standard. Sample preparation for the measurements started with an annealing up to 800 °C and the combustion residue was dissolved in a 3:1 mixture of hydrochloric and nitric acid at 200 °C. Quantitative analysis of the samples was performed using an Agilent 7890A gas chromatograph coupled to an Agilent 5975C mass selective detector. A Restek Rxi-1MS column (30 m × 0.25 mm × 0.25 mm) was used for the measurements. Three analytical standards (2,4-toluene-diamine, 2,4-dinitrotoluene and 2-methyl-5-nitroaniline) were employed for the analysis of the samples.

### Catalytic tests: hydrogenation of 2,4-dintitrotoluene

The catalytic hydrogenation process was carried out in a Büchi Uster picoclave reactor with continuous stirring (1000 rpm). The hydrogenation of dinitrotoluene in methanol was tested at four different temperatures (303 K, 313 K, 323 K and 333 K). The H_2_ pressure was 20 bar, which was kept constant by a pressure regulator. The initial concentration of 2,4-DNT in methanol was 50 mmol L^−1^ to which 0.1 g of catalyst was added. The samples were collected after 5, 10, 15, 20, 30, 40, 60, 120, 180, and 240 min. As internal standard, 5.0 µL nitrobenzene was added to 1.00 mL sample. The concentration of the main product (TDA) and the detected by-products and intermediates were measured using an Agilent 7890A gas chromatograph and an Agilent 5975C mass selective detector. The volume of the injected sample was 1 μl at a 200:1 split ratio, while the input temperature was set at 473 K. Helium was used as a carrier gas at a constant flow rate of 2.28 ml/min. The oven temperature was initially set at 323 K and then heated at 10 K/min to 523 K, where it was maintained for 3 min.

The efficiency of the catalysts was determined by calculating the conversion (X%) of DNT using the following equation (Eq. 1):

1 $$ X\%  = \frac{{consumed\;\varvec{n}_{{DNT}} }}{{initial\;\varvec{n}_{{DNT}} }} \cdot 100 $$

The yield (Y%) of TDA was calculated based on the following equation (Eq. 2):

2 $$ Y\%  = \frac{{formed\;\varvec{n}_{{TDA}} }}{{theoritical\;\varvec{n}_{{TDA}} }} \cdot 100 $$

where *n*_TDA_ and *n*_DNT_ are the molar amounts of the compounds.

Catalyst selectivity (S%) was calculated using DNT conversion and TDA yield as follows (Eq. 3):

3 $$ S\%  = \frac{Y}{X} \cdot 100 $$

## Results and discussion

### Characterization of the NiFe_2_O_4_/N-BCNT magnetic catalyst support

**Figure 1:**
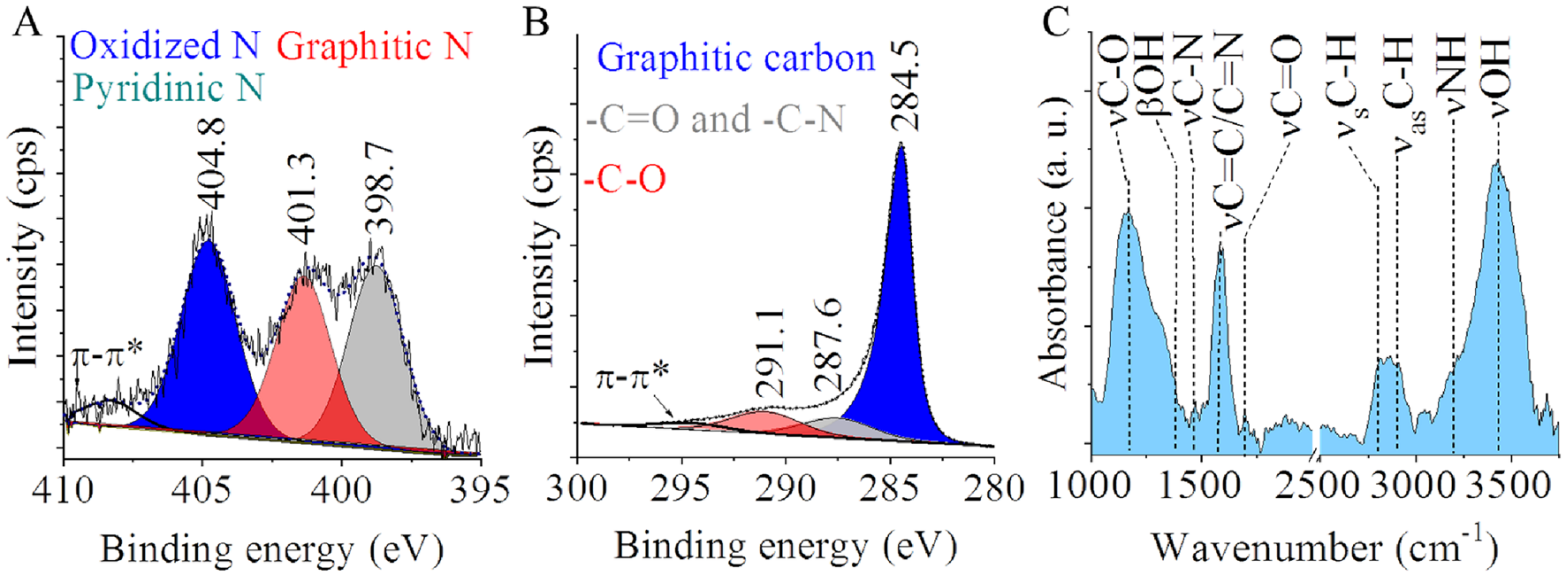
Deconvoluted N 1 s (**A**) and C 1 s (**B**) bands on the XPS spectrum, and FTIR spectrum (**C**) of the N-BCNTs.

The N-BCNT samples were characterized by XPS for identification of the incorporated nitrogen forms and the oxygen containing functional groups. (Fig. 1). The incorporation of the nitrogen atoms into the structure of N-BCNTs occurred due to the nitrogen content of the carbon precursor (N-butylamine). On the deconvoluted N 1 s band three peaks were identified at 404.8 eV, 401.3 eV, and 398.67 eV binding energy which are attributed to the oxidized pyridinic N atoms (pyridine oxide), graphitic and pyridinic nitrogen atoms, respectively (Fig. 1A). On the deconvoluted C 1 s band those peaks were identified, which belong to the -C=N and –C–N bonds at 287.6 eV, moreover the peak of the –C–O bonds was found also, which originates from the oxygen containing functional groups (hydroxyl, carboxyl, carbonyl etc.) (Fig. 1B).

**Figure 2:**
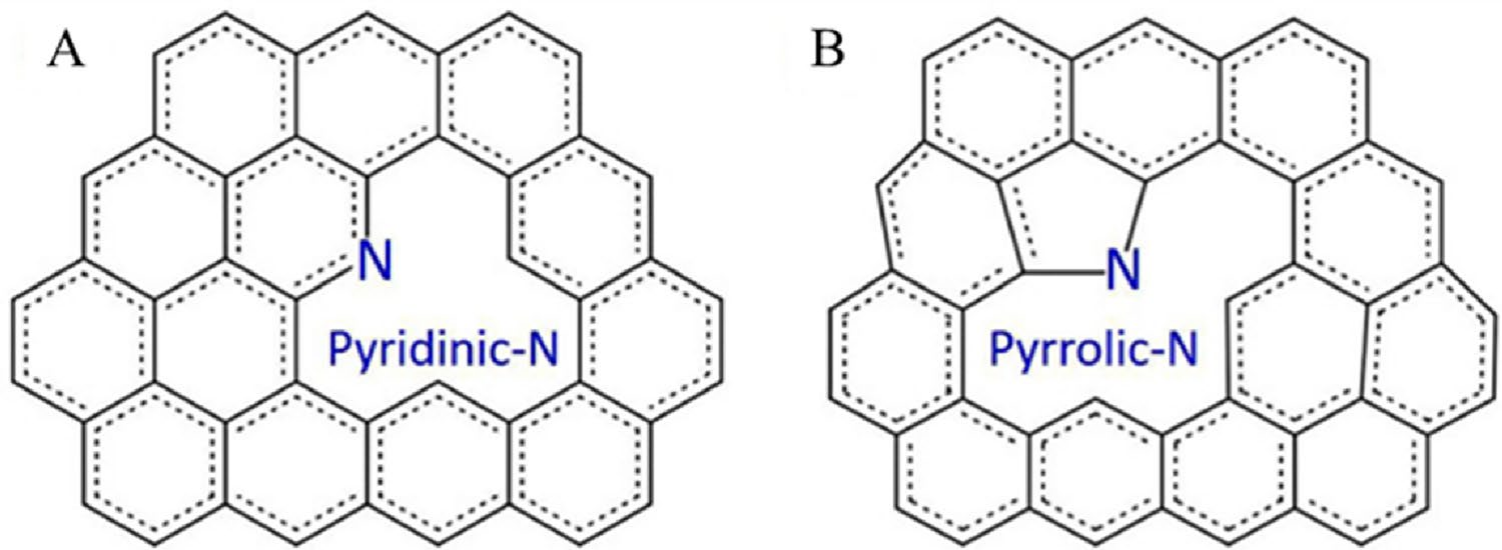
Schematic illustration of the nitrogen atoms and the vacancies in the carbon lattice, pyridinic (**A**) and pyrrolic (**B**) types^26^.

The incorporation of nitrogen atoms (mainly the pyridinic and pyrrolic and types) into the honeycomb-like lattice are accompanied with the structural defects (vacancies, bonding disorders, and non-cyclized structures) which creates high disorder of the structures (Fig. 2A and B)^26^.

FTIR spectroscopy was additionally invoked to identify the functional group on the surface of the N-doped carbon nanotubes (Fig. 1C, SI Fig. 1). A broad band can be seen on the infrared spectra centered at 3430 cm^−1^ wavenumber, which correspond to the stretching vibrations of O–H bonds (νOH), which originates from hydroxyl and carboxylic groups and adsorbed water^27^. The peak of the νOH bond is convoluted with the vibration band of the νNH bonds, which is identified as shoulder (around 3200 cm^−1^) on the hydroxyl bond vibration. The peak at 1396 cm^−1^ can be associated with O–H bending in carboxyl groups. The band at 1703 cm^−1^ with low intensity belongs to νC = O vibrations in carbonyl or carboxyl groups^28^. The C–O stretching resulted absorption band at 1170 cm^−1^, owing to the presence of the hydroxyl groups^29^. The peak at 1595 cm^−1^ is belong to vibrations of aromatic C=C and C=N bonds^30,31^. Other vibration band is also located on the spectrum, which is characteristic for the N–H bonds in-plane deformations, and it is found at 1530 cm^−1^ with low intensity, and overlaps with other bands^32^. The peak at 1460 cm^−1^ is belong to the C–N stretching vibrations^33^. The absorption band of the aliphatic C–H bonds are located at 2790 cm^−1^ and 2899 cm^−1^^34^.

The presence of the oxygen containing functional groups, namely the -OH and -COOH on the carbon supports is very useful. These groups can be deprotonated and lead to negatively charged surface which contribute to the anchoring of the Fe^3+^ and Ni^2+^ as precursor ions can decompose to NiFe_2_O_4_ spinel nanoparticles on the catalyst supports during preparation. These groups are identifiable by evaluating the C 1 s peak on the XPS spectrum also (Fig. 1B).

**Figure 3:**
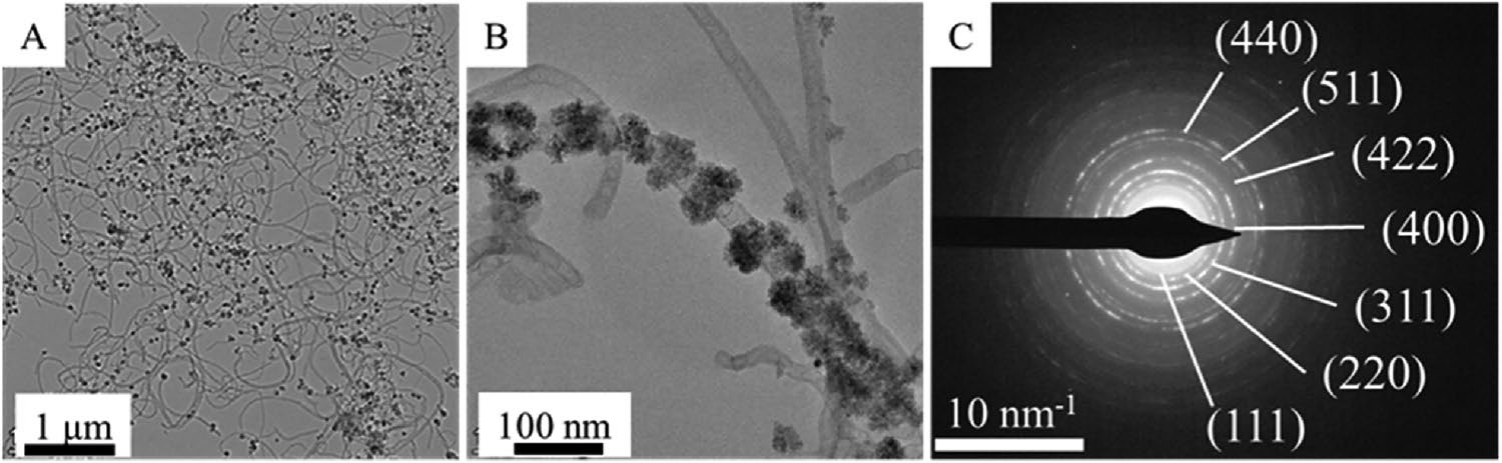
TEM images (**A**, **B**), and SAED (**C**) of the NiFe_2_O_4_ nanoparticles deposited on the N-BCNT surface.

On the TEM images of the NiFe_2_O_4_/N-BCNT catalyst support, it is visible, that the nanotubes were richly decorated by nickel ferrite nanospheres (Fig. 3A). The mentioned nickel ferrite spheres build up from individual nanoparticles which are smaller than 10 nm (SI Fig. 2). These nanospheres were in the range of 40 ± 13 nm in diameter for the prepared NiFe_2_O_4_/N-BCNT system (SI Fig. 3.). Moreover, it can be seen at higher resolution, that the ferrite nanoparticles crystallized in such an orientation that they embrace the cylindrical shell of the carbon nanotubes (Fig. 3B). Based on these, we expect that, in addition to sorption interactions, a strong mechanical connection has also developed between the ferrite and the nanotube. As a result, a stable catalyst support was obtained, from which the ferrite nanoparticles expected not to be eliminated during applying in the catalytic reaction. Selected area electron diffraction (SAED) was used to prove the presence of nickel ferrite phase (Fig. 3C). Typical patterns of the diffraction rings were identified as reflections of the nickel ferrite (PDF 10-0325).

**Figure 4:**
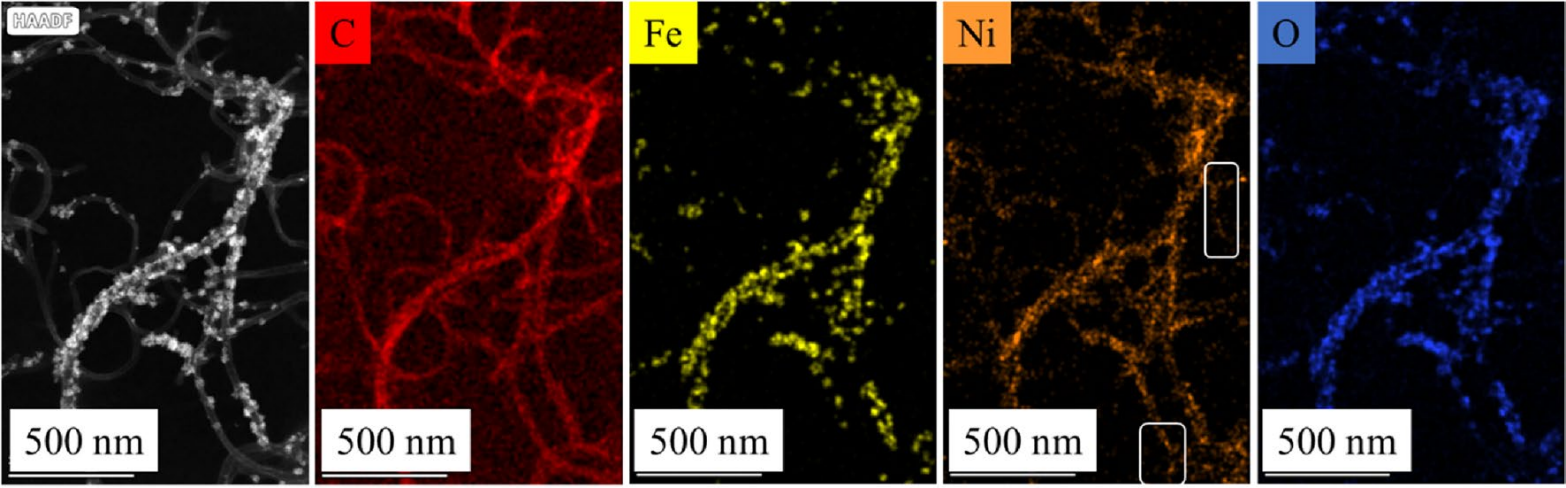
Element maps of the prepared NiFe_2_O_4_/N-BCNT system.

On the element maps of the magnetic catalyst supports, iron, nickel, and oxygen are shown on surface of the carbon nanotubes (Fig. 4). Another interesting thing can be observed on the elemental map of iron and nickel, because next to nickel, the sign of iron is not visible everywhere. It follows that nickel is also present independently from iron, in the form of nanoparticles, on the surface of the nanotubes. The nickel and iron is also able to play a role of catalyst promoter in the hydrogenation of aromatic nitro compounds.

### Characterization of the platinum decorated NiFe_2_O_4_-NBCNT supported magnetic catalyst

Specific surface area (SSA) is one of the most important properties of a catalyst that can affect its adsorption capacity and performance. Thus, the SSA of the prepared catalyst was determined from carbon dioxide adsorption measurements using the Dubinin-Astakhov isotherm, resulting in a surface area of 63.43 m^2^/g. This is significantly smaller compared to the specific surface area of a Pt-containing catalyst supported on activated carbon, which is 829 m^2^/g^35^. An average activated carbon’s pores are made up of more than 95% micropores, which negatively affect material transport and, consequently, the reaction rate during a catalytic process^36^. In contrast to activated carbons, carbon nanotubes primarily have mesopores, which provide easier and faster access for reactant molecules to the surface.

The particle morphology of the platinum containing NiFe_2_O_4_/N-BCNT catalyst was investigated using HRTEM (SI Fig. 4.). The TEM images also show the spherical morphology of the ferrite particles, which retained their shape after platinum deposition. Based on these results, it can be concluded that the ferrite particles did not undergo any structural damage during the high-energy ultrasonic treatment. Therefore, a stable nanostructured catalyst support was obtained in this sense.

**Figure 5:**
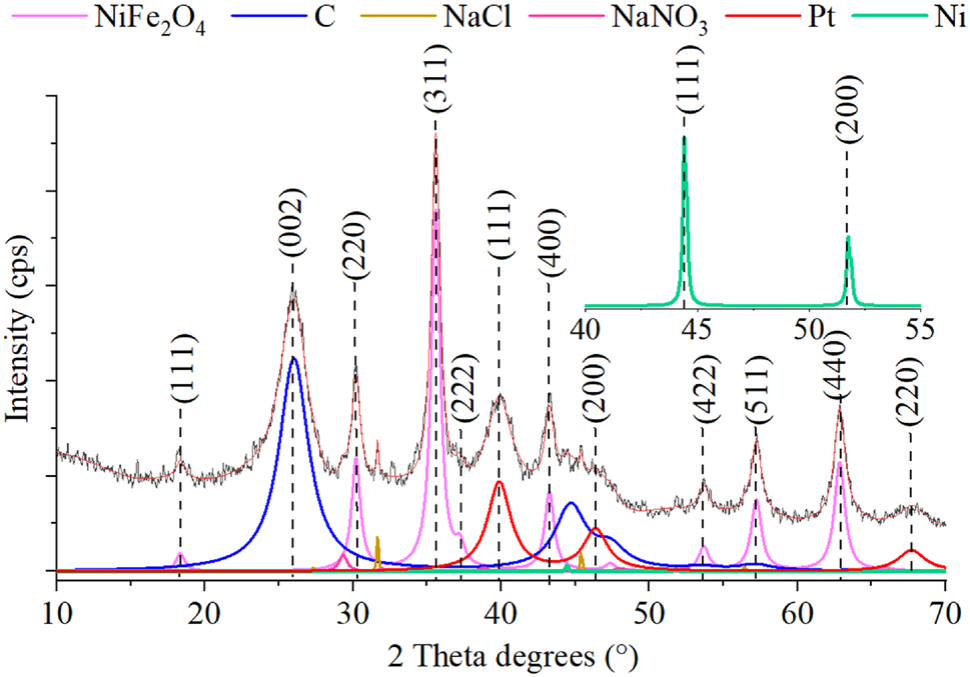
XRD pattern of the Pt/NiFe_2_O_4_/N-BCNT catalyst.

XRD analysis was applied for identification of the different crystalline phases formed on the N-BCNT support (Fig. 5). On the Rietveld refined XRD pattern (111), (220), (311), (222), (400), (422), (511), and (440) reflections were identified, which belongs to the nickel ferrite spinel, these peaks are located at 18.3°, 30.1°, 35.5°, 37.2°, 43.1°, 53.5°, 57.1° and 62.7° two theta degrees (PDF 10-0325). The peaks which are typical for the Pt nanoparticles are (111), (200), and (220) and have been found on the diffractogram of the catalyst at 39.8, 46.4, and 67.2 two Theta degrees, respectively (PDF 04-0802). The carbon nanotubes resulted reflections at 24.7° (002), and 37.8° (100) two theta degrees (PDF 23-0064). Similarly to the element mapping (Fig. 3), nickel was identified in the catalyst support, and the corresponding reflections, (111) and (200), found at 44.4° and 51.7° two theta degrees (PDF 04-0850). Moreover, sodium nitrate was also found in the sample, according to reflections at 23.0° (012), 29.3° (104), 31.2° (006), 35.8° (110), 39.3° (113), 43.1° (202), 47.4° (018), 48.4° (116), 56.4° (122), 57.4° (214), 60.6° (208), 62.9° (125), 64.7° (300), 69.0° (217) and 72.7° (128) two theta degrees (PDF: 00-036-1474). The presence of the NaNO_3_ can be explained by the reaction between the sodium ions (from the sodium acetate) and nitrate ions (from the iron- and nickel precursors) during the coprecipitation of the NiFe_2_O_4_ nanoparticles. Sodium chloride was also identified in low quantity in the sample as it can be seen reflections at 31.7° (200) and 45.4° (220) 2 Θ degrees (PDF 78-0751). The formation of sodium chloride can be explained by the reaction of chloride ion from the dihydrogen hexachloroplatinate complex (the precursor of platinum) with sodium ions.

The Pt decorated magnetic N-BCNT catalyst contained 39.7 wt% nickel ferrite, next to 3.0 wt% platinum, 0.2 wt% nickel, 0.7 wt% sodium chloride and 2.5 wt% sodium nitrate. We also studied whether an alloy phase was formed between nickel and platinum. Comparing the corresponding measured unit cell parameters of Pt and Ni, a = 3.9135 and a = 3.5272, to the theoretical values of a = 3.9231 and a = 3.5238, a significant difference is revealed in Pt, which is not related to Ni, since the metallic radius of Pt is 1.36 pm while Ni is 1.24 pm. Alloying of such metals inadvertently leads to increase in unit cell (a-value) if smaller radius metal is alloyed by larger radius metal, and vice versa. Thus, in our case Pt is not alloyed, but in contrary, the a-value difference arises from the strain related to the ultra-nanocrystalline (5 nm average size) nature of the particles, and the intrinsic association with Ni indicated by HRTEM X-ray mapping should be related to the Ni-in-Pt precipitation process.

**Figure 6:**
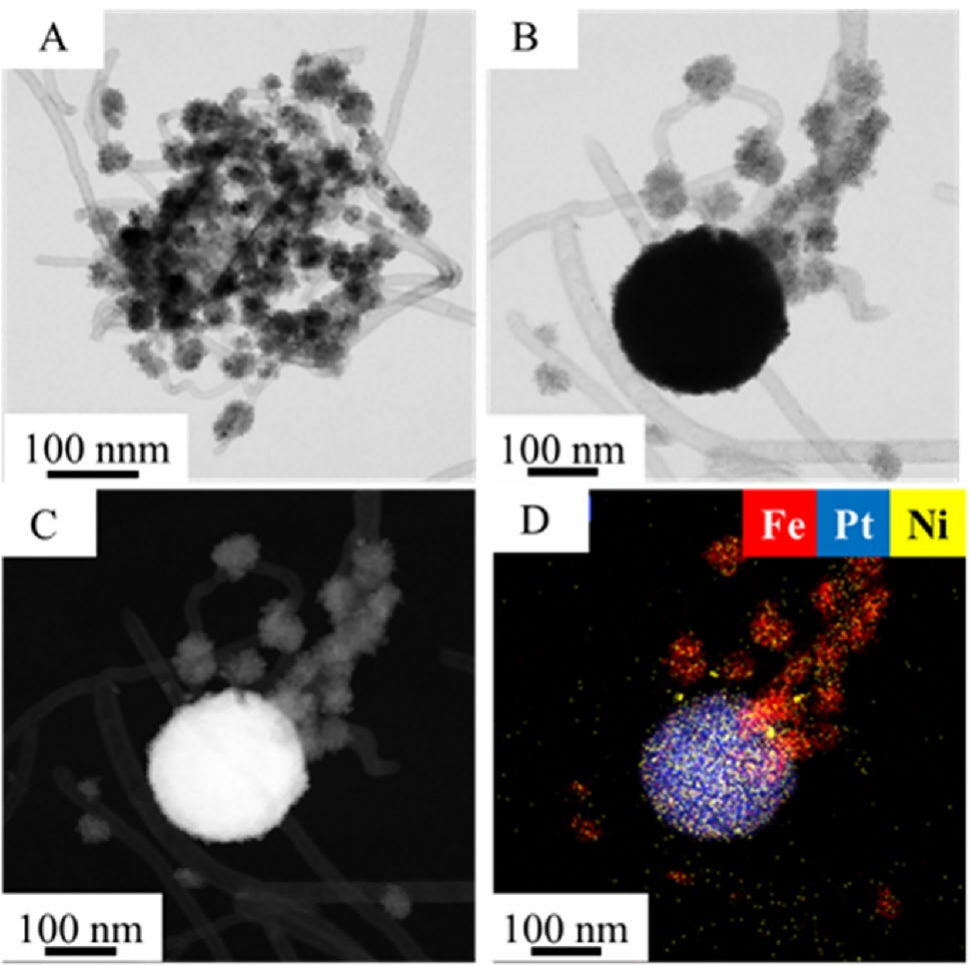
HRTEM images (**A**, **B**) and element maps (**C**, **D**) of the prepared Pt/NiFe_2_O_4_/N-BCNT catalyst.

On the HRTEM images of the Pt/NiFe_2_O_4_/N-BCNT catalyst (Fig. 6A,B), higher (d ~ 200 nm), spherical particles are seen (Fig. 5B). Based on the element mapping, it can be stated that these spherical particles build up from iron, nickel, and platinum (Fig. 6C,D). This suggests a synergistic effect between the metals that contributes to the increased catalytic activity during the hydrogenation of aromatic nitro compounds^37^. In the case of bimetallic catalysts, the synergistic effect is partly due to electron transfer (electron effect). Since the electronegativity of Ni atom (1.91) is smaller than that of Pt atom (2.28), some electrons of Ni atoms are taken away by Pt atoms, causing electron transfer between the two metals^38^. Due to the electron transport mentioned above, the Pt particle shows less affinity for the π-electron system of aromatic nitro compounds, which affects the adsorption of reactant molecules on the metal surface. Since, the adsorption of aromatic nitro compounds on the catalytically active metal surface can take place in parallel orientation (by the phenyl group) or through the oxygen of the nitro group (perpendicular orientation). The modes of adsorption of nitrobenzene on Pt(111) and Ni(111) surfaces have been investigated previously. Sheng and colleagues used density functional theory (DFT) calculations to determine the binding energies and showed that nitrobenzene is more likely to bind through its phenyl group on the Pt(111) surface^39^. It was found that the phenyl group could be adsorbed on the surface via the formation of C-Pt bonds, due to the strong overlapping of the partially empty d-band of Pt with the pz orbitals of the phenyl group, the calculated adsorption energy was − 1.71 eV. Such a strong interaction between phenyl group and Pt(111) resulted in the rehybridization of the carbon orbitals from sp2 to sp3 and as a consequence, chemisorption dominated. The binding energy was also studied in cases when the nitrobenzene molecules were cross-linked to the platinum atoms via the nitro group, and it was found that the average distance of Pt-O bond would be 2.27 Å, and the calculated adsorption energy is very low (− 0.66 eV), which would make nitrobenzene unfavorable to adsorb via nitro-group. In the case of Ni(111) surface, similar nitrobenzene orientation was reported by Mahata et al.^40^ based on DFT simulations, namely the parallel adsorption of the NB molecules over a catalyst surface is preferable over vertical adsorption. Adsorption through the phenyl group can also lead to the formation of over hydrogenated byproducts, when the aromatic ring becomes saturated with hydrogen. This can be avoided by using bimetallic catalysts, due to the electronic effect of the promoter metal detailed above.

**Figure 7:**
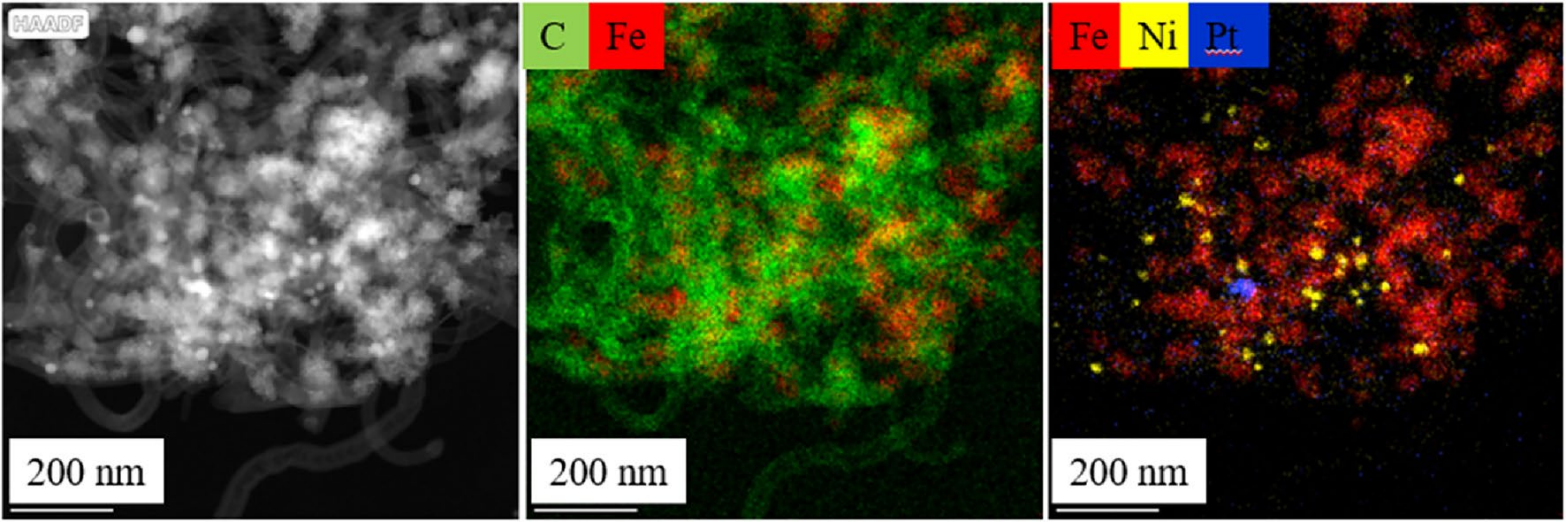
Element maps of the Pt/NiFe_2_O_4_/N-BCNT catalyst.

Furthermore, it can also be observed that where nickel is present, iron is not always found based on the element maps (Fig. 7). Thus, it can be concluded that the nickel particles can also be found separately and independently on the surface of the nanotubes, as we also demonstrated in the case of the XRD measurement (Fig. 5). In addition to NiFe_2_O_4_ phase (Ni^2+^ in the spinel), the catalyst also contains elemental nickel (Ni^0^), in which case synergism can also be assumed during the catalytic process. Similar results were reported before, and the synergy between the Ni^2+^ and Ni^0^ were experimentally verified, because the Ni^0^ species activates and dissociates H_2_ to obtain active hydrogen, and Ni^2+^ adsorbs and polarizes N=O group via the lone electron pairs in oxygen^41^.

### Catalytic performance of the magnetic Pt/NiFe_2_O_4_/N-BCNT catalyst

First, the platinum-free magnetic support was tested in the hydrogenation of 2,4-dinitrotoluene. The test showed that the noble metal-free catalyst support also exhibits activity in the synthesis of TDA. In our previous work, we have also succeeded in producing magnetic catalyst supports that showed activity during the hydrogenation of DNT^42^. The NiFe_2_O_4_/N-BCNT support alone resulted in a 70.5% conversion of DNT at 333 K, while the TDA yield was 4.97% after 240 min of reaction. However, due to the low TDA yield, it is essential to deposit a noble metal on the surface of the catalyst support.

In addition to the main product (TDA), two semi-hydrogenated intermediates, 4-amino-2-nitrotoluene (4A2NT) and 2-amino-4-nitrotoluene (2A4NT), were identified during the hydrogenation carried out in the presence of the catalyst support . These results are consistent with the literature^43,44^. Janssen et al. were the first to study a reaction mechanism whereby 2,4-dinitrotoluene is converted to 2,4-diaminotoluene via two parallel reaction pathways^45,46^. The o- and p-nitro groups are converted to amino groups in different reactions. While the o-nitro group is converted directly, the p-positioned hydroxylamine is converted to the corresponding amino compound via an intermediate consecutive reaction. Neri et al. carried out the hydrogenation of 2,4-dinitrotoluene to 2,4-toluylenediamine using a 5% Pd/C catalyst^1,47^. They found that the hydrogenation of 2,4-DNT to 2,4-TDA can be described by a complex reaction mechanism involving only three relevant intermediates, namely 4HA2NT, 4A2NT, and 2A4NT. Condensation products (i.e., azoxy, azo, and hydrazo compounds) were not formed during the reaction, but the presence of 2-amino-4-hydroxyaminotoluene and another azoxy compound was observed.

**Figure 8:**
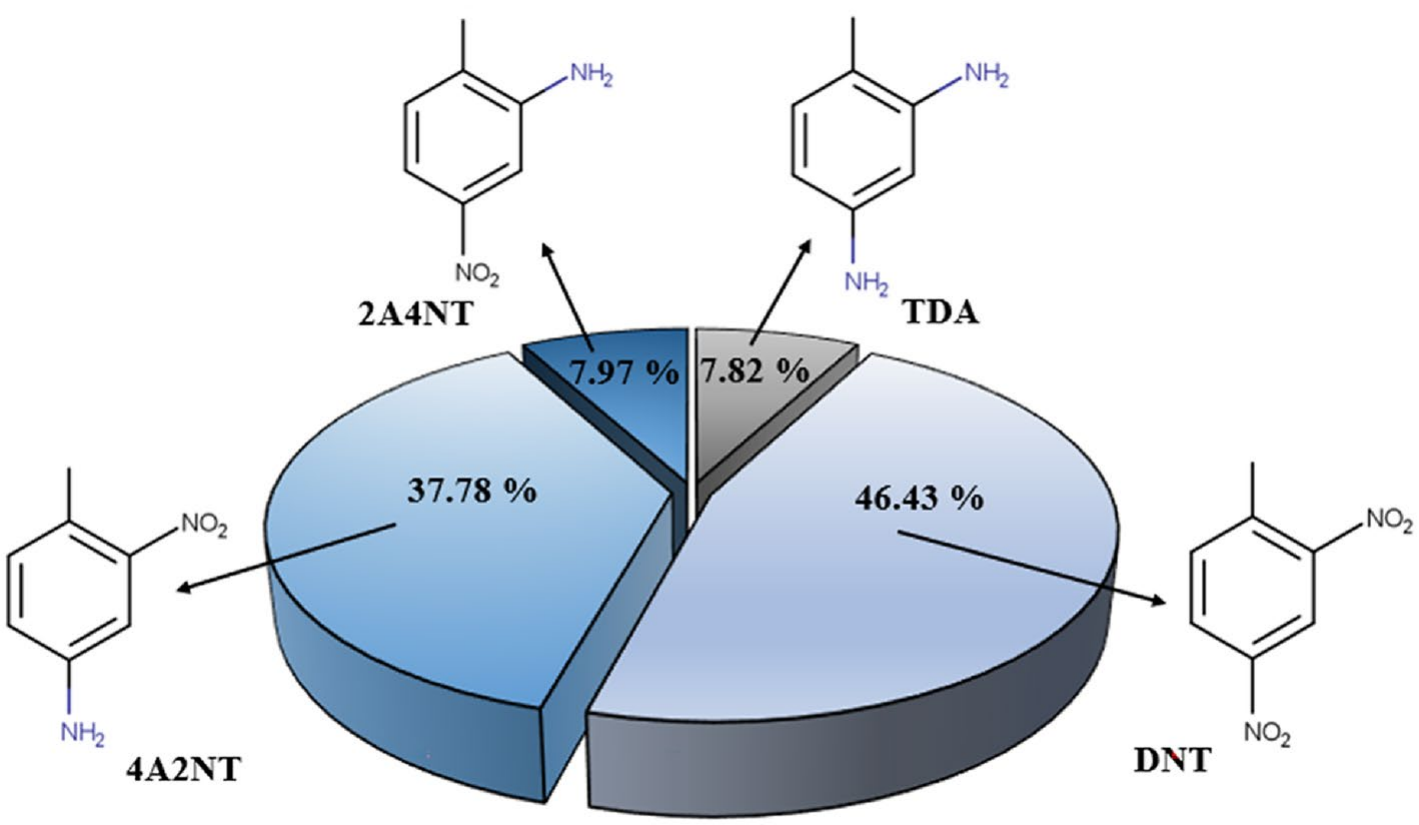
Proportion of each component in the hydrogenation 2,4-DNT carried out in the presence of the prepared NiFe_2_O_4_/N-BCNT support at 330 K within four hours.

During the catalytic hydrogenation of the magnetic catalyst support, a higher proportion of intermediates 4A2NT (37.78%) and 2A4NT (7.97%) were formed in addition to the main product (TDA) (Fig. 8).

**Figure 9:**
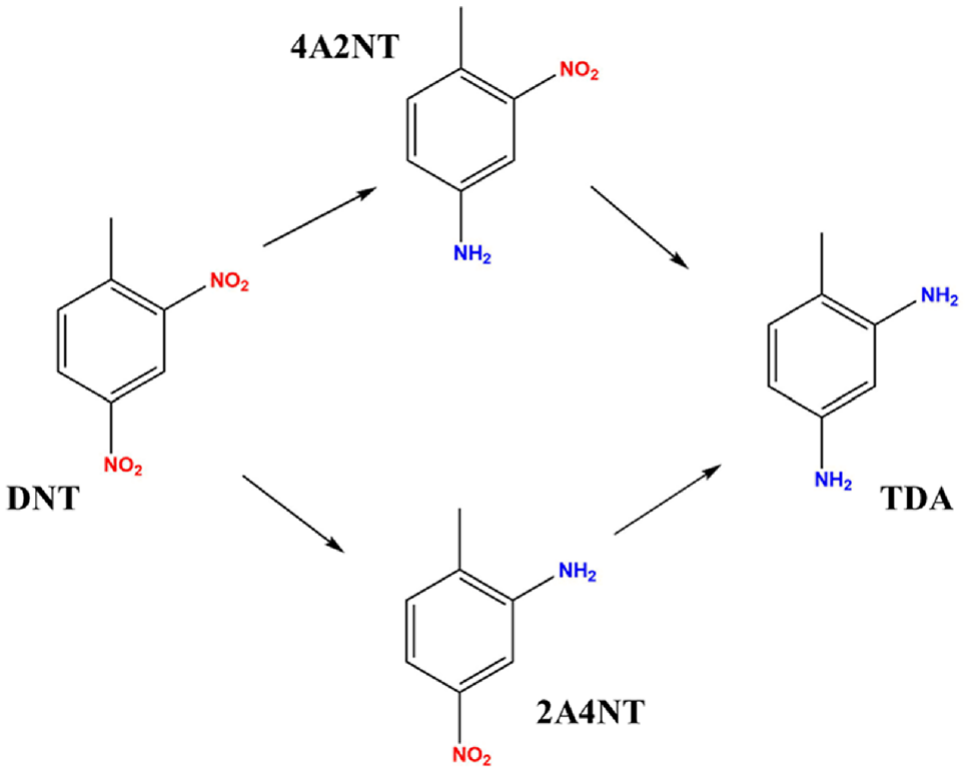
Proposed mechanism of DNT hydrogenation to produce TDA.

From these results, it can be concluded that the NiFe_2_O_4_/N-BCNT support is suitable for the synthesis of the two intermediates without noble metals. The results of the catalytic tests and previous findings suggest the following possible reaction mechanism (Fig. 9):

Musolino et al.^48^ investigated the selective liquid-phase hydrogenation of 2,4-DNT on different catalysts to produce the corresponding 2,4-nitroarylhydroxylamines. They found that the temperature and the nature of the support also affected the distribution of the products.

The catalytic activity of the platinum-containing NiFe_2_O_4_/N-BCNT supported catalyst was tested at four different temperatures (303, 313, 323, and 333 K), and the temporal change in DNT concentration was measured. The reaction parameters are shown in Table 1.

**Table 1:** Reaction parameters used during hydrogenation.

Reaction parameters	
Temperature (K)	313–333
Pressure (bar)	20
Solvent	Methanol
Amount of solvent (ml)	150
Initial c_DNT_ (mmol L^−1^)	50
Catalyst mass (g)	0.1
NiFe_2_O_4_ (wt%)	39.7
Pt (wt%)	3.0
Ni (wt%)	0.2

**Figure 10:**
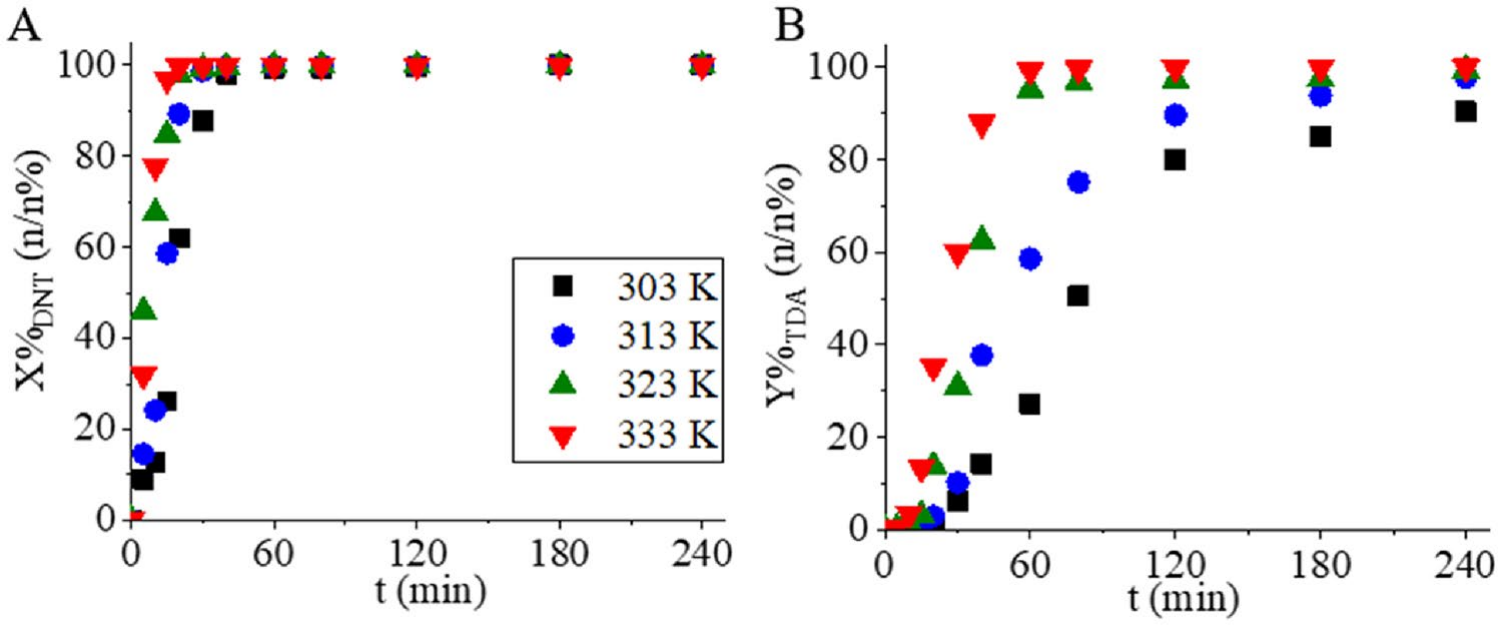
DNT conversion (**A**) and TDA yield (**B**) as a function of reaction time at various temperatures. The measurements were carried out in the presence of the magnetic Pt/NiFe_2_O_4_/N-BCNT catalyst.

The conversion was maximal (~ 100 n/n%) at all four temperatures (Fig. 10A). Complete conversion was achieved with the Pt/NiFe_2_O_4_/N-BCNT magnetic catalyst within 40 min at 333 K, but full conversion was achieved after two hours of hydrogenation at all temperatures. For the Pt/NiFe_2_O_4_/N-BCNT catalyst, the TDA yield did not significantly change with increasing reaction temperature; the 30 K temperature difference resulted in only a ~ 14% improvement in yield over the four-hour hydrogenation period. The maximum TDA yield was 99.9 n/n% at 333 K and 20 bar hydrogen pressure (Fig. 10B).

The MIRA21 model^25^ was used to compare the catalyst with other catalytic systems in the literature. The database contains 58 different catalysts tested in DNT hydrogenation, covering the full range of catalysts. The model describes the parameters used to characterize catalyst performance and ranks and classifies catalysts. The Pt/NiFe_2_O_4_/N-BCNT catalyst has a MIRA21 score of 10.95, ranking as the 12th best catalyst.

**Figure 11:**
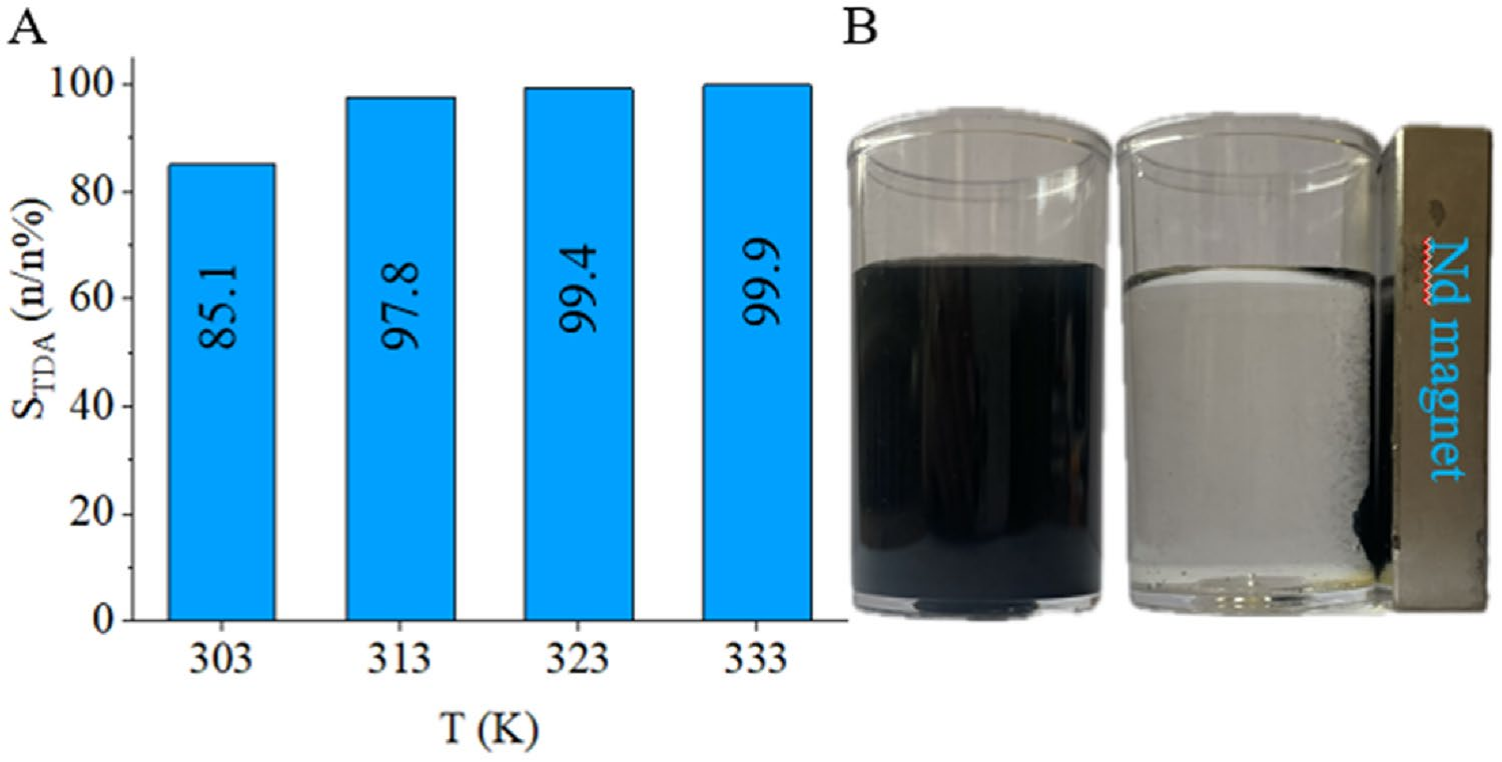
Maximum selectivity of TDA at four reaction temperatures applying the magnetic Pt/NiFe_2_O_4_/N-BCNT catalyst and separation from the reaction medium using a neodymium magnet.

In addition to 2,4-TDA as the main product, GC measurements also detected the partially hydrogenated intermediate products 4-amino-2-nitrotoluene (4A2NT) and 2-amino-4-nitrotoluene (2A4NT). Using the Pt/NiFe_2_O_4_/N-BCNT catalyst, the total amount of transition product was further converted to the target product after 120 min of hydrogenation at a reaction temperature of 333 K. Product selectivity for 2,4-TDA was found to be above 99 n/n% during the reaction at 333 K (Fig. 11A).

Due to the functional groups, the hydrophilicity of the nanotube surface can be improved and a higher dispersibility can be achieved, which is important for the preparation of CNT-supported catalysts. The catalyst is well dispersible in methanol solution, and the stable catalyst dispersions could be efficiently separated from the reaction medium by an external magnetic field (Fig. 11B).

**Figure 12:**
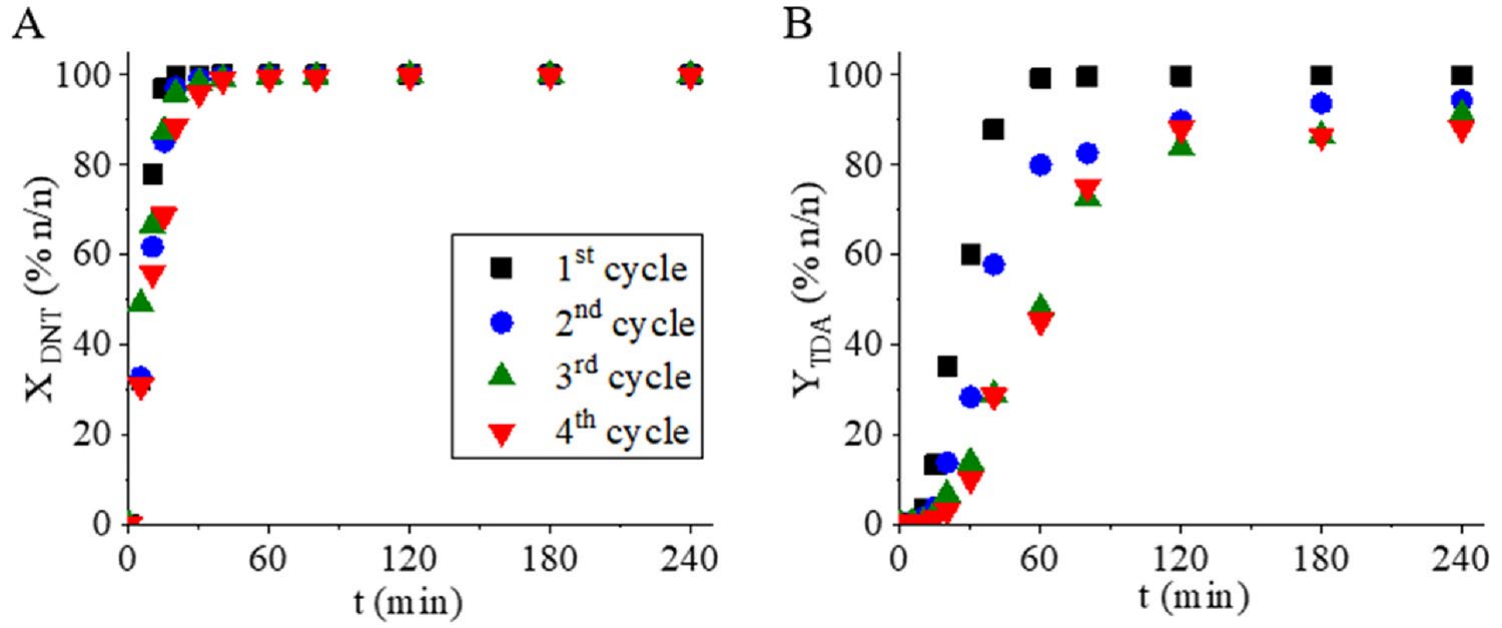
Variation of 2,4-DNT conversion and 2,4-TDA yield as a function of hydrogenation time in reuse tests of the Pt/NiFe_2_O_4_/N-BCNT catalyst.

The reusability and stability of catalysts are important factors for industrial applications. For this reason, the reusability of the Pt/NiFe_2_O_4_/N-BCNT catalyst was investigated (Fig. 12). The catalyst was tested in four cycles at 333 K and under identical reaction conditions (Table 1). After the reaction was completed, the catalyst was easily separated using a magnet, not regenerated, only washed with methanol and reused in the next cycle. The DNT conversion remained stable and did not decrease significantly even after four cycles. There was no significant decrease in TDA yields observed over two cycles, with TDA yield > 94 n/n% after 240 min of hydrogenation. However, a significant decrease was observed in the fourth reuse test, with a maximum TDA yield of 88.2 n/n% after four hours of hydrogenation. The total amount of the two semi-hydrogenated intermediates, 2A4NT and 4A2NT, was further converted to the target product at the end of the hydrogenation.

ICP-OES measurements were carried out on the unused catalyst and the catalyst used four times. The platinum content of the fresh catalyst was 3.03 wt%, which decreased slightly to after the fourth cycle (2.41 wt%). Thus, the Pt/NiFe_2_O_4_/N-BCNT catalyst can be successfully used in dinitrotoluene hydrogenation, but regeneration of the system is required between cycles to avoid precious metal leaching and activity loss.

## Conclusions

NiFe_2_O_4_/N-BCNT supported magnetic catalyst successfully prepared with remarkable catalytic activity. The magnetic catalyst support was synthesized by a modified coprecipitation method. Platinum nanoparticles were deposited on the surface of ferrite particles by a rapid, relatively simple, and efficient sonochemical method, resulting in a readily applicable catalytically active system. It was confirmed that the noble metal-free catalyst support also possesses catalytic activity. However, the presence of platinum dramatically increased the catalytic performance. Conversion was excellent at all four studied temperatures. Under the applied reaction conditions, the optimal reaction temperature was 333 K, as complete conversion (100%) was achieved within 40 min using the developed Pt/NiFe_2_O_4_/N-BCNT catalyst. After a hydrogenation time of 2 h, a 99% yield and selectivity of TDA was reached. The total amount of semi-hydrogenated intermediate products was further transformed into the target product. During the reuse tests, DNT conversion remained stable and did not decrease significantly even after four cycles, with TDA yields above 88% throughout the tests. In addition, the magnetic catalyst can be easily recovered from the reaction medium by the action of an external magnetic field, which can significantly reduce operating costs and catalyst loss during separation. Thus, a catalyst with superb properties was achieved which can applicable in industrial settings to carry out catalytic hydrogenation processes.

### Supplementary Information

Supplementary Information.

## Data Availability

Data is provided within the manuscript and supplementary information files.
