# Supplementary material for: Nickel ferrite decorated noble metal containing nitrogen-doped carbon nanotubes as potential magnetic separable catalyst for dinitrotoluene hydrogenation
Source: Sci Rep. 2024 Jul 2;14:15156. doi: 10.1038/s41598-024-66066-1 (PMC11219928; doi:10.1038/s41598-024-66066-1)
Supplement: Supplementary file 1 — Supplementary Information. [file 41598_2024_66066_MOESM1_ESM.docx]

Nickel ferrite decorated noble metal containing nitrogen-doped carbon nanotubes as potential magnetic separable catalyst for dinitrotoluene hydrogenation

Viktória Hajdu^1,2^, Emőke Sikora^1^, Gábor Muránszky^1^, Ferenc Kristály^3^, Zoltán Kaleta^4,^, Miklós Nagy^1^, Béla Viskolcz^1,2^, Béla Fiser^1,5,6, ,*^, László Vanyorek^1,*^

*^1^Institute of Chemistry, University of Miskolc, Miskolc-Egyetemváros, 3515, Hungary*

*^2^Higher Education and Industrial Cooperation Centre, University of Miskolc, Miskolc-Egyetemváros, 3515, Hungary*

*^3^Institute of Mineralogy and Geology, University of Miskolc, 3515, Miskolc-Egyetemváros, Hungary*

*^4^Department of Organic Chemistry, Semmelweis University, 1092 Budapest, Hungary*

*^5^Ferenc Rakoczi II Transcarpathian Hungarian College of Higher Education, Beregszász, 90200, Ukraine*

*^6^Department of Physical Chemistry, Faculty of Chemistry, University of Lodz, 90-236, Lodz, Poland*

**Correspondence: bela.fiser@uni-miskolc.hu & laszlo.vanyorek@uni-miskolc.hu*

**Supplementary Information**


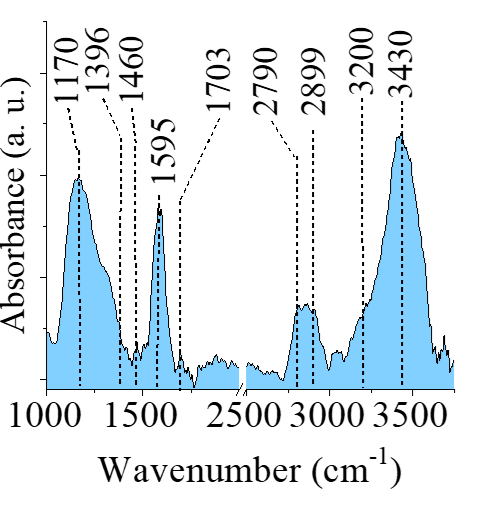


SI Fig 1: FTIR spectrum of the N-BCNTs


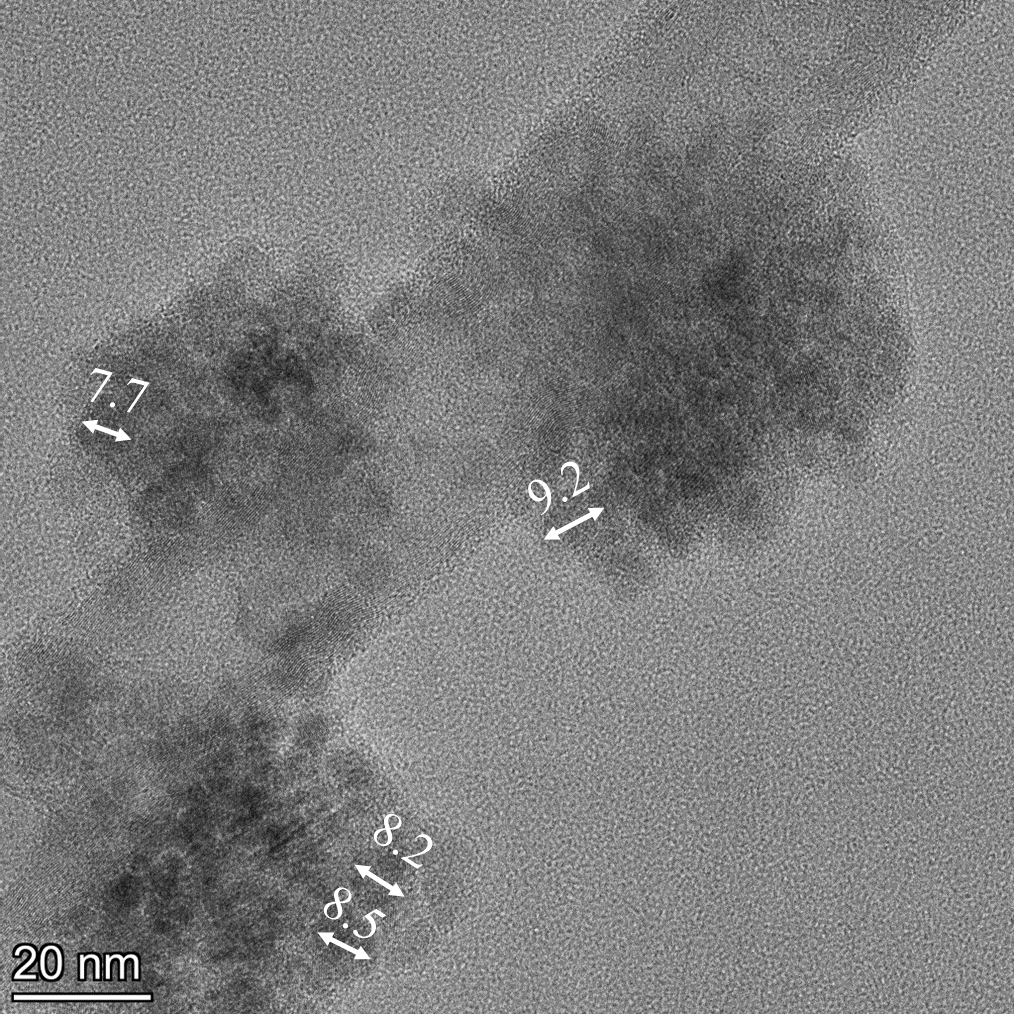


SI Fig. 2: TEM image of the nickel ferrite nanoparticles


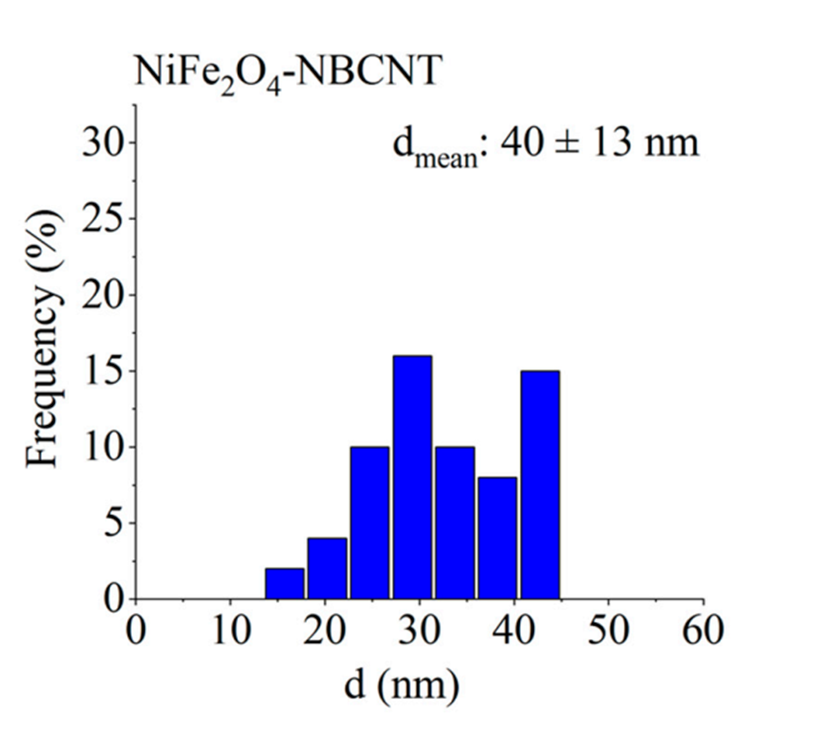


SI Fi. 3: Particle size distribution of the NiFe_2_O_4_ nanospheres on the N-BCNT support


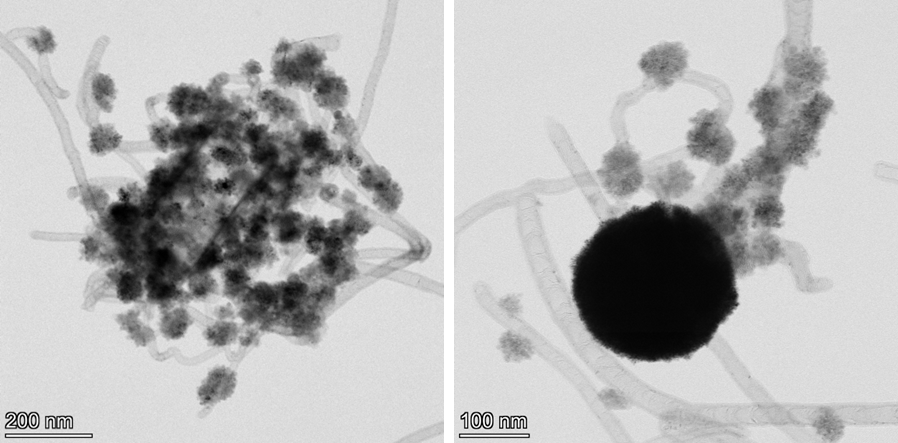


SI Fig. 4: TEM images of the Pt/NiFe_2_O_4_/N-BCNT catalyst
